# Analysis of ankle inversion sprain injury mechanism from accidental injury cases captured in televised basketball matches

**DOI:** 10.1186/1757-1146-7-S1-A30

**Published:** 2014-04-08

**Authors:** KM Chan, Sophia CW Ha, Daniel TP Fong, KM Chan

**Affiliations:** 1 Division of Biomedical Engineering, Department of Electronic Engineering, The Chinese University of Hong Kong, Hong Kong; 2 Department of Orthopaedics and Traumatology, Prince of Wales Hospital, Faculty of Medicine, The Chinese University of Hong Kong, Hong Kong; 3 School of Sport, Exercise and Health Sciences, Loughborough University, Leicestershire LE11 3TU, UK

## Introduction

The aim of this study was to use model-based image matching method (MBIM) to study ankle inversion sprain injury mechanism from basketball cases. MBIM can be used to understand the injury mechanism quantitatively by analyzing the three-dimensional human motion [1].

## Methods

An ankle inversion sprain injury occurred in a televised basketball match was found from the internet. The videos were transformed into uncompressed AVI image sequence by using Adobe Premiere Pro (CS4, Adobe Systems Inc, San Jose, California). Then the image sequences were synchronized and rendered into 1-Hz video sequences by Adobe After-Effects (CS4, Adobe Systems Inc). 3-dimension animation software Poser 4 and Poser Pro Pack (Curious Labs Inc, Santa Cruz, California) were used to perform the matching part. Virtual environment was built according to the real dimensions of a basketball court and it was manually matched to the background for each frame in every single camera view. The skeleton model from Zygote Media Group Inc. (Provo, Utah) was used to match with the athlete. The segment dimensions were adjusted according to the subject’s height. The skeleton matching started with the hip, thigh, shank segment and then distally matched the foot and toe segments frame by frame. The ankle time histories were input into Microsoft Excel (Microsoft Office, Microsoft, US) to calculate the velocity-related information.

## Results

The peak inversion in this case lies within the range (48°-142°) obtained in previous studies [1]. Different from previous studies [1], plantarflexion is found at the time of peak ankle inversion during the injuring motion.

**Table 1:** Peak value of the ankle angles and velocities (inversion, internal rotation and plantarflexion) were shown in the table below. The lowest row indicates the duration of the ankle sprain injury.

Max. Inversion angle (deg)	110
Max. Inversion velocity (deg/sec)	2916.

Time of peak inversion (sec)	0.24

Max. Internal rotation angle (deg)	56

Max. Internal rotation velocity (deg/sec)	551

Time of peak internal rotation (sec)	0.52

Max. Plantarflexion angle (deg)	32

Max. Plantarflexion velocity (deg/sec)	580

Time of peak plantarflexion (sec)	0.40

Whole duration (sec)	0.52s

## Conclusion

The analysis of basketball ankle inversion ligamentous sprain case was done and compared with previous studies. Since no basketball cases have been analyzed before, so more basketball cases should be analyzed by MBIM in order to understand the real injury mechanism.

## References

[B1] Fong DTP Ha SCW Mok KM Chan CWL Chan KM Kinematics analysis of ankle inversion ligamentous sprain injuries in sports - five cases from televised tennis competitions The American Journal of Sports Medicine 2012 40 11 2627 2632 10.1177/0363546512458259 22967824

